# A retrospective, cross-sectional analysis of the dental status and needs of patients taking vascular endothelial growth factor (VEGF) antagonists

**DOI:** 10.1007/s00784-025-06230-7

**Published:** 2025-02-22

**Authors:** Alexander Raucci, Katherine France

**Affiliations:** 1 Department of Oral Medicine, University of Pennsylvania School of Dental Medicine, Philadelphia, PA USA; 2 240 South 40th St, Schattner 234, Philadelphia, PA 19104 USA

**Keywords:** VEGF antagonists, Dental status, Medically complex dentistry, DMFT

## Abstract

**Objectives:**

There is growing literature related to the dental effects of biologic agents. However, little research has evaluated the dental needs of these patients and limited data is available on patients taking vascular endothelial growth factor (VEGF) antagonists.

**Materials and methods:**

This retrospective cross-sectional study analyzed patients taking VEGF antagonists and their respective dental treatments from 2017 to 2023. Patient demographics, decayed, missing, and filled tooth (DMFT) status, and treatment details were recorded and evaluated descriptively. DMFT from this population was compared to patients taking other biologic agents using t-tests.

**Results:**

Twenty-three patients taking VEGF antagonists received dental treatment. Periodontal treatments were the most common (*n* = 18, 78%), followed by restorative treatments (*n* = 17, 74%). DMFT data for patients taking VEGF antagonists were not significantly different than values for patients taking other biologic agents, except for patients taking TNF-α Inhibitors, who had significantly fewer missing teeth than patients taking VEGF antagonists (3.88 vs. 8.10, *p* = 0.01).

**Conclusions:**

The necessity of dental treatment in patients taking VEGF Antagonists is still unknown. Periodontal and restorative treatments were the most common modalities with no complications observed from treatment. While preventive periodontal treatment is routinely recommended, high usage of targeted periodontal and restorative treatments is seen in this population.

**Clinical relevance:**

Minimal evidence exists to date on the delivery of and need for routine dental treatment among patients taking VEGF antagonists. This paper presents a first estimate of dental needs in this population.

**Supplementary Information:**

The online version contains supplementary material available at 10.1007/s00784-025-06230-7.

## Introduction

Biologic Agents (BA) are a class of drugs defined as “a substance that is made from a living organism or its products and is used in the prevention, diagnosis, or treatment of cancer and other diseases.” [1] These drugs include monoclonal antibodies that have the following suffices: human (“mab”), humanized (“zumab”), chimeric or mouse–human (“ximab”), and variant fusion proteins (“cept”) [2]. Biologic agents are used across specialties of medicine in a wide variety of applications including the treatment of inflammatory conditions and immunotherapy for advanced malignancies. The therapeutic effects of these agents depend on the pathway targeted.

Vascular Endothelial Growth Factor (VEGF) antagonists are a subset of biologic agents that target the formation of new blood vessels through inhibition of VEGF, a naturally-occurring factor promoting angiogenesis [3]. These drugs are “a type of antisense oligonucleotide and a type of gene expression inhibitor” that bind to the receptor for VEGF and prevent the formation of blood vessels in tumor cells and other environments of rapid growth [4]. VEGF antagonists have therefore been employed in the treatment and management of cancer. Bevacizumab, for example, a VEGF antagonist with over 15 years of market data, has been found to decrease the growth and metastasis of several cancer types, including metastatic colorectal, metastatic breast, non-small-cell lung, renal cell carcinoma, glioblastoma, ovarian, and cervical cancers [5]. Along with their oncologic effects, VEGF antagonists have also been shown to greatly decrease the angiogenesis and vascular leakage associated with pathogenic causes of blindness and vision loss, such as Diabetic Retinopathy (DR) and Age-Related Macular Degeneration (AMD) [6]. 

Along with the increase of VEGF antagonists in the treatment of cancers and visual degenerative disorders, there is increasing study into their negative effects, specifically on wound healing. VEGF has often been studied for its crucial role in the rehabilitation and regrowth of tissues. One study found that, although VEGF alone does not promote bone regeneration, it works synergistically with osteogenic factors (specifically bone morphogenic protein (BMP) 4) to “increase recruitment of mesenchymal stem cells, to enhance cell survival, and to augment cartilage formation in the early stages of endochondral bone formation.” [7] It has also been documented in rat models that inflammatory factors (i.e. Prostaglandin E_2_) produced in response to drug-induced lesions promoted the expression VEGF and increased angiogenesis, showing a cross-relationship between the inflammatory response and recruitment of VEGF to expedite wound healing [8]. 

Given the importance of VEGF and of angiogenesis to wound healing, concerns have been raised that VEGF antagonists could have negative consequences to patients post-operatively and/or following traumatic injury, with confirmatory evidence documented in multiple studies. In an interventional study on mouse muscle-derived stem cells, it was found that a VEGF antagonist, Soluble Fms-like Tyrosine Kinase-1 (sFlt1), significantly inhibited BMP2-induced bone formation, while VEGF promoted bone formation in conjunction with osteogenic factors, such as BMP2 and BMP4 [9]. Similarly, in a meta-analysis of case reports, literature reviews, and reports to the United States Food and Drug Administration of 144 patients, mean visual acuity dropped by 6.4 lines with severe visual loss after anti-VEGF injection, and the “overall risk of ocular vascular events following a VEGF antagonist injection was 0.108% in the general population and 2.61% in the diabetic population.” [10] These findings are believed to be caused by VEGF’s role as a vessel dilator through the stimulation of nitric oxide synthesis, meaning blocking this pathway could cause damage to the retina due to decreased retinal perfusion caused by low ophthalmic systolic pressure [10]. 

The same studies show a possible link between VEGF antagonists and reduced bone regeneration and mucosal tissue repair, which could result in an increased rate of periodontal destruction through decreased bone regeneration and a higher rate of complications after procedures. In addition, patients taking VEGF antagonists suffer medical compromise from their underlying conditions and may therefore have difficulty maintaining oral hygiene or seeking required dental treatment. There is currently little literature showing the effects of these drugs on the incidence of dental disease necessitating treatment, although one study found that the failure and complication rate after dental implant placement was similar between medically complex and healthy groups [11]. 

This descriptive study aimed to address this gap through an evaluation of the dental treatment needs and experiences in patients taking VEGF antagonists across four categories (Restorative, Periodontal, Endodontic, and Oral Surgical procedures). Through characterization of the dental treatment experiences of this population, this study begins to elucidate the rate at which patients taking VEGF antagonists experience dental treatment needs. Secondarily, and by comparing the overall dental status of this population to that of patients taking other classes of biologic agent and to a general adult population, this study provides a first estimation of differences in treatment experience across categories of agent. Moreover, our study attempts to provide data to build insight into the safe delivery of dental treatment in patients on VEGF antagonists, a topic that has very limited evidence to date in medical literature.

## Materials and methods

In this retrospective cross-sectional study, we collected all available data on dental treatments rendered, and complications following these treatments, on patients taking VEGF antagonists over a 6-year period at University of Pennsylvania School of Dental Medicine (Philadelphia, Pennsylvania).

We included all data on adult patients (> 18 years old) taking VEGF antagonists in any outpatient clinic at the University of Pennsylvania from July 1, 2017, through July 1, 2023. Our study period was defined to start at the inception of an updated electronic health record system (axiUm, Exan, Las Vegas, NV) in our center. Beginning our study period after axiUm was implemented standardized reliability of our data. Patients were identified if they had a VEGF antagonist included in their medications list using either generic or brand name. A list of VEGF antagonists searched for can be found in the Appendix. Dental treatment types were collected based on their respective Code on Dental Procedures and Nomenclature (CDT) codes [12]. Details on data gathered are available in the Appendix. Given that this study included all patients taking VEGF antagonists across an educational institution, these procedures were either performed by licensed dentists alone, residents, or dental students under supervision of licensed dentists.

Patients were excluded from the study if they ended VEGF antagonist treatment prior to the data collection start date (July 1, 2017) or if they were found on chart review not to be taking a VEGF antagonist. Given the possible and incompletely characterized lasting effects of VEGF antagonist treatment, including via continued inflammation and increased rate of infection which may modify dental treatment needs, data was included for the entire treatment period after starting the agent, even if they stopped treatment during the study timeframe. We collected demographic information for all patients, including sex (male, female, or other according to the options available in the employed medical record), race (White, Asian, Black, Native Hawaiian or Other Pacific Islander, and unreported), year of birth, first date of dental treatment, most recent date of dental treatment, start date of VEGF antagonist treatment (if available), and end date of VEGF antagonist treatment (when applicable).

We reviewed included patient charts, documenting each dental treatment rendered while taking the VEGF antagonist, according to CDT codes. We also reviewed patient charts for any complications to healing following procedures. Decayed, Missing, Filled Teeth (DMFT) data was collected from each patient. All data was obtained directly from the electronic medical record system and organized using standardized secure Microsoft Excel spreadsheets (Microsoft Corporation, Redmond, WA), which were created before data collection started.

Data collection forms were approved by all study team members before study inception and formally reviewed during extraction. All data was extracted by a single researcher (AR) and independently verified (KF). No identifying data was collected. Demographic and treatment data were gathered collectively and compared descriptively. Due to our sample size, we summarized the data based on VEGF antagonists as a collective, as opposed to disaggregating the individual drugs used in treatment. Treatment experience was compared according to the number of study patients in active treatment for each year to control for needs that remained unidentified if patients did not present for care. We compared DMFT data between patients on VEGF antagonists to data previously obtained on patients taking three other classes of Biologic Agent (TNF-alpha antagonists, interleukin inhibitors, and lymphocyte modulators, data on request) using two-sided T-Tests to identify differences between these subsets of biologic agent. We also compared this data to publicly available DMFT data through the Center of Disease Control (CDC) from 2017 to March 2020 [13]. We defined significance as *p* < 0.05 in each case.

### Compliance with ethical standards

The study was deemed exempt by the University of Pennsylvania Institutional Review Board (Protocol 853738). All data was de-identified, and informed consent was not required per institutional review board review.

## Results

From July 1, 2017, to July 1, 2023, 23 patients treated with VEGF Antagonists were treated at our center. The mean (SD) year of birth was 1957 (13.4), with 12 and 11 patients being male and female, respectively (Table 1). Although race was unreported for most patients (*n* = 12, 52%), at least four patients were White, five patients were African American, one identified as both White and African American, and one reported their race as Other. Patients were treated with 3 VEGF antagonists: Bevacizumab, Aflibercept, and Ranibizumab. Twelve patients were taking Bevacizumab (Avastin/prAvastin, Genentech), while five patients were taking Aflibercept (Eylea, Bayer/Regeneron), and seven patients were taking Ranibizumab (Lucentis, Genentech). One patient was prescribed both Aflibercept and Ranibizumab non-concurrently during the study period.

**Table 1:** Demographics of included patients taking VEGF antagonists treated between July 1, 2017 and July 1, 2023, according to data available in electronic health records

	All Agents	Bevacizumab	Aflibercept	Ranibizumab
**Year of Birth**,** y**,** mean**	1957	1964	1953	1948
**Birth Year Range**	1934–1981	1947–1981	1934–1962	1939–1957
**Total # Patients**	23*	12	5	7
**Sex**,** no.**				
Male	12*	5	3	5
Female	11	7	2	2
**Race**,** no.**				
White	5*	1	1	2
Black or African American	6*	2	1	2
Other	1	1	0	0
Unreported	12	7	3	3
**Disease Treated**				
Primary Cancer**	6	6	0	0
Metastatic Cancer***	3	3	0	0
Ocular Degeneration****	13	3	4	5
Not Stated	1	0	0	1
**Other Medical Conditions (%)**				
Hypertension	12			
GERD	7			
High Cholesterol	6			
Diabetes	5			
Heart Murmur	3			
Iron Deficiency	3			
Rheumatoid Arthritis	1			
Osteoarthritis	1			

*Some totals do not sum to 23 because one patient was taking both aflibercept and ranibizumab during portions of the study period, and one other patient identified as both White and Black or African American

**To the best of our knowledge, these patients were prescribed VEGF Antagonists for treatment of primary cancers without metastasis, including cervical, colon, rectal, liver, uterine, and kidney cancers

*** To the best of our knowledge, these patients were prescribed VEGF Antagonists for treatment of metastatic cancers, including metastatic basal cell carcinoma, rectal adenocarcinoma, and metastatic colon cancer

****To the best of our knowledge, these patients were prescribed VEGF Antagonists for treatment of ocular degenerative diseases, including diabetic retinopathy, retinal vasculitis, macular vein thrombosis, and macular degeneration

The reasons for VEGF antagonist treatment included ocular diseases including macular degeneration and glaucoma, as well as cervical, colon, lung, ovarian, renal, and liver cancers. We categorized treatment reasons as: Primary Cancer, Metastatic Cancer, and Ocular Degeneration (Table 1). The most common reason was ocular degeneration (*n* = 13). Patients had many shared comorbid medical conditions, with hypertension being the most common (*n* = 12), followed by gastroesophageal reflux disease (GERD) (*n* = 7), high cholesterol (*n* = 6), and diabetes (*n* = 5) (Table 1). The number of patients on active VEGF antagonist treatment increased per year over the study period and is summarized in Table 2. The years 2017 and 2023 include only 6 months of data given that collection started and ended, respectively, on July 1 of these years.

**Table 2:** Number of patients overall and on each VEGF inhibitor receiving dental treatment per year

	2017	2018	2019	2020	2021	2022	2023
**All Agents**	5	8	7	10	13	11	6
**Bevacizumab**	1	2	1	4	6	6	3
**Aflibercept**	1	2	3	3	4	2	2
**Ranibizumab**	4	5	4	4	3	3	1

**Fig. 1:**
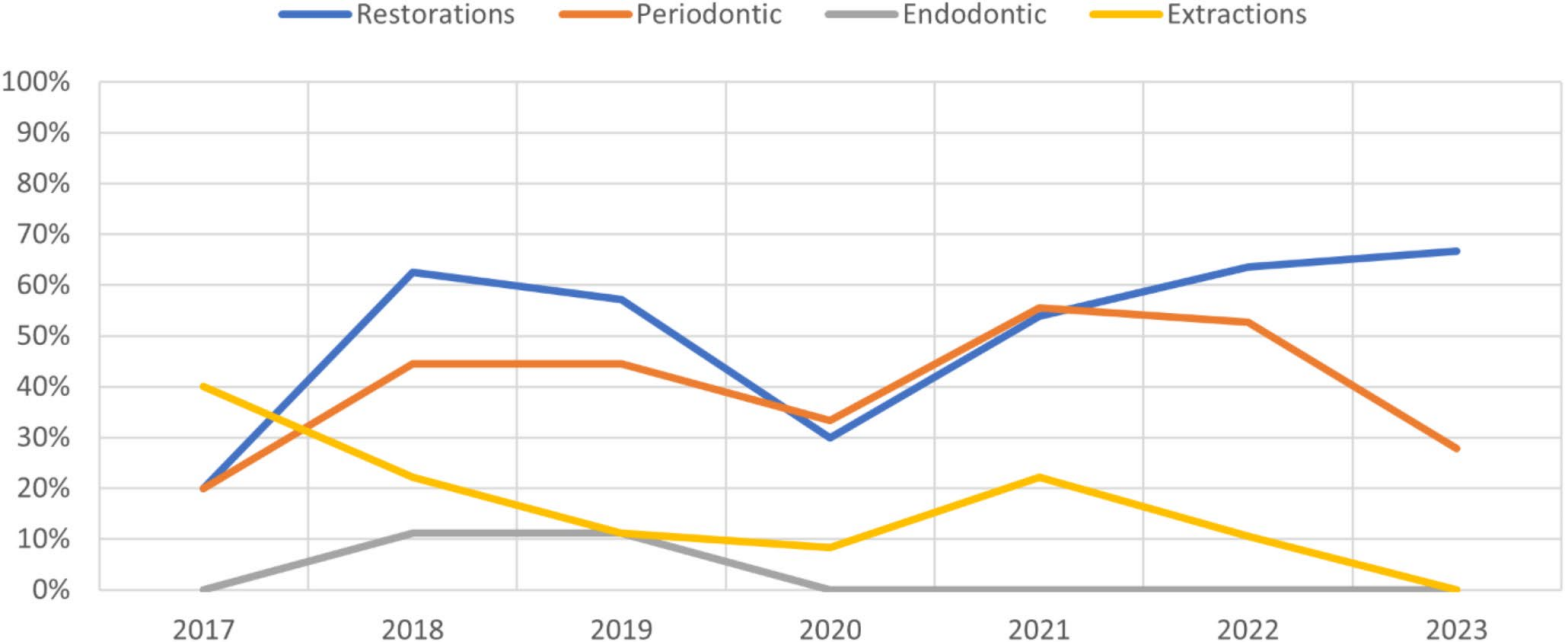
Dental treatment use by patients taking VEGF Antagonists during the study period, shown as a percent of active patients receiving restorative, periodontal, endodontic, and oral surgical treatments each year

Of the four dental treatment categories evaluated (Restorative, Periodontal, Endodontic, and Oral Surgery), the percentage of patients taking VEGF Antagonists who received each treatment is summarized in Fig. 1 and counts of each treatment can be found in Table 3.

**Table 3:** Distribution of dental treatments received by patients taking VEGF antagonists over study period, detailing number of treatments received and percent, calculated as the percent of patients receiving active treatments per year receiving the treatment type in question

Total # per Procedure Type (%)	2017	2018	2019	2020	2021	2022	2023	AVG
Restorative	1 (20.0)	5 (62.5)	4 (57.1)	3 (30.0)	7(53.9)	7 (63.6)	4 (66.7)	4.4 (50.5)
Periodontal	1 (20.0)	4 (44.4)	4 (44.4)	4 (33.3)	10 (55.6)	10 (52.6)	5 (27.8)	5.4 (39.7)
Endodontic	0 (0)	1 (11.1)	1 (11.1)	0 (0)	0 (0)	0 (0)	0 (0)	0.3 (3.2)
Oral Surgery	2 (40)	2 (22.2)	1 (11.1)	1 (8.3)	4 (22.2)	2 (10.5)	0 (0)	1.7 (16.3)

**Fig. 2:**
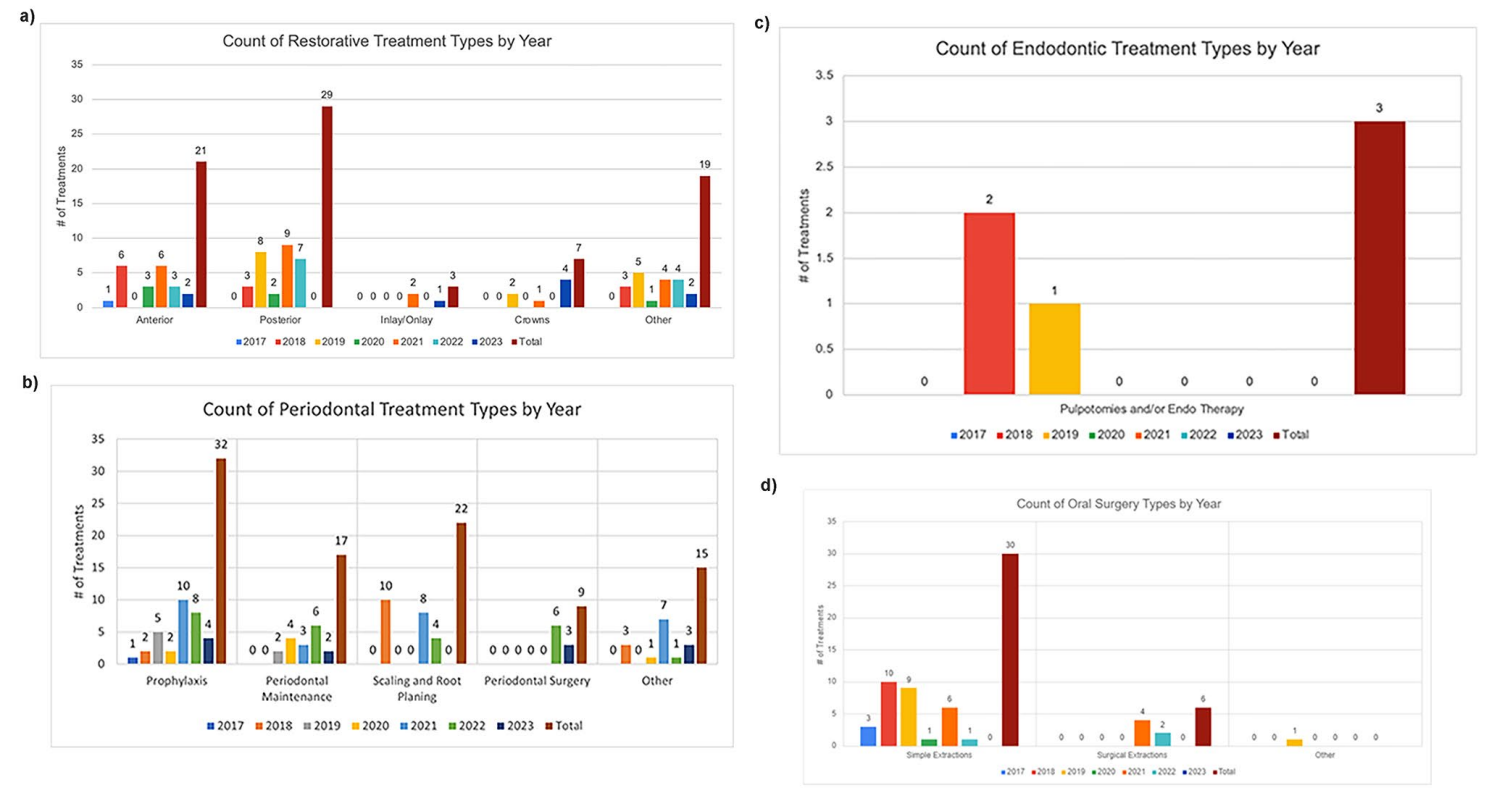
Dental treatment received by patients taking VEGF antagonists by type of dental treatment and by year shown as count of treatments received each year per treatment type divided into (A) restorative treatment, (B) periodontal treatment, (C) endodontic treatment, (D) oral surgical treatments over the study period 2017–2023

The count of each type of Restorative treatment by year and their associated totals is summarized in Fig. 2.a. Patients most commonly required anterior and posterior restorations, including 21 anterior restorations (26.6% of all restorative treatments) and 29 posterior restorations (36.7% of restorations). Only one patient received inlay or onlay treatments during the study timeframe. The “Other” category consists of a few common procedure types, most frequently complete dentures, both mandibular (D5120) and maxillary (D5110), as well as temporary restorations.

The count of each type of Periodontal treatment by year and their associated totals is summarized in Fig. 2.b. Prophylaxis and scaling and root planing treatments were the most frequent periodontal procedures. Patients received 32 total prophylaxis cleanings and 22 scaling and root planing treatments, which account for 33.7% and 23.2% of all periodontal procedures, respectively. Implant placement was observed in our population and is included as periodontal surgery. All periodontal surgeries included were completed without evidence of post-surgical complication in associated clinical records. The “Other” category for periodontal treatments consists of ancillary treatments including re-evaluations after scaling and root planing (SRP) and guided tissue regeneration.

The count of each type of Endodontic treatment by year and their associated totals is summarized in Fig. 2.c. The only Endodontic treatment types rendered during the study time frame were pulpotomies and/or primary endodontic therapies (*n* = 3 total). These treatments were also completed without evidence of complication.

The count of each type of Oral Surgical treatment by year, and their associated totals, is summarized in Fig. 2.d. The majority of oral surgeries were simple extractions (*n* = 30/37, 81.1%). The “Other” category was intended to capture alveoplasties, soft and hard tissue biopsies, and other adjunctive treatments. However, only one patient received a surgical treatment outside of extraction, which was an alveoplasty in conjunction with tooth extractions (D7310) with no evidence of complication after the procedure. Notably, on review of all patient charts, there was no evidence of delayed wound healing, complications after procedures, or other unexpected response to treatment after oral surgery in any member of this cohort.

The averages for the Decayed, Missing, and Filled Teeth (DMFT) data from each patient on VEGF antagonists are listed in Table 4. This data was compared to previously obtained average DMFT data for patients at our center taking three other classes of Biologic Agent (TNF-α antagonists, interleukin inhibitors, and lymphocyte modulators) between 2017 and 2022 (Table 4). Patients taking VEGF inhibitors were found to have statistically fewer missing teeth than those taking TNF-α inhibitors. To compare with available national DMFT data as published by the US CDC, the published metrics for the 65–74 year old age group most closely aligned with the average age in our study (average cohort age = 66.2 years old, age range 42–89). On one-sided t-test, decayed and missing data were not significantly different between our published data and this national average. However, filled data was significantly different (VEGF average = 6.30 vs. national average = 9.00, *p* = 0.01).

**Table 4:** Decayed, missing, and filled tooth indices for the combined group of patients taking VEGF antagonists included in this analysis (top row) as compared to metrics found in patients taking other classes of biologic agent in the same institution (B or T cell, interleukin, and TNF-α inhibitors) with comparisons to VEGF agent data using paired t-test results, *p* = 0.05, highlighted cell denotes a statistically significant difference. The last row compares DMFT data for the VEGF antagonist cohort to data from the Center for Disease Control from 2017 to March 2020 for patients 65 to 74 years old using a one sample t-test, *p* = 0.05

	Decayed		Missing		Filled	
**VEGF Antagonists**,** Avg.**	1.55		8.10		6.30	
	**Decayed**,** mean**	**T-Test result (p)**	**Missing**,** mean**	**T-Test result (p)**	**Filled**,** mean**	**T-Test result (p)**
Lymphocyte modulator*	2.04	0.35	7.92	0.47	7.58	0.22
Interleukin inhibitor*	1.68	0.39	5.40	0.10	7.53	0.15
TNF-α inhibitor*	1.53	0.38	3.88	0.01	6.22	0.45
**C** **DC Data for 65–74 Year Olds**	0.20	1.41	5.60	1.48	9.00	0.01

*The comparator patients taking other classes of biologic agents (*n* = 247 total, lymphocyte modulator *n* = 27, interleukin inhibitor *n* = 75, TNF-α inhibitor *n* = 151) were being treated with the medications in question for a variety of indications classified as arthritic conditions (*n* = 115), colitis (*n* = 48), dermatologic diseases (*n* = 28), and others including rheumatologic, neurologic, pulmonary, ENT, oncologic, and multiple indications. These patients were born between 1934 and 1998 (mean 1969) with 153 female, 94 male, and 1 patient of unknown gender. Full analysis of this cohort is available on request

## Discussion

To date, the oral health of patients taking VEGF antagonist treatment has not been studied extensively outside of a few case reports and case series, mostly related to the possibility of developing osteonecrosis. For example, a retrospective study of patients with metastatic colorectal carcinoma who had been treated with Bevacizumab long-term (> 6 months) between January 2006 and November 2013 found that the drug promoted bone hypoperfusion within humeral and femoral heads, leading to rare cases (4/1000 patients) of osteonecrosis [14]. This finding has been further substantiated by a few case studies of patients taking VEGF antagonists who experienced Osteonecrosis of the Jaw (ONJ) [15–17]. Another study looking at wound healing of palatal mucosa in rat models found that giving dimethyloxalylglycine (DMOG), which inhibits the degradation of hypoxia inducible factor-1 alpha (HIF-1α) and consequently increases the expression of angiogenic factors, increased wound contractions in the palatal mucosa [18]. This provides evidence on the interplay between angiogenic processes and wound healing as may occur in patients taking VEGF inhibitors and illustrates the need to investigate the oral health status of this patient population.

Aside from these findings, however, few studies have looked at the development of dental needs or delivery of treatment in patients on VEGF antagonist treatment. This study addresses this gap through an evaluation of the dental treatment experience in patients on these drugs being treated at the same large academic center. Our patients on active VEGF antagonist treatment frequently required restorative treatments (*n* = 17, 74%), with most of these treatments being direct restorations, as are most frequently required in cases of dental caries. While there is no established expected rate of restorative treatments in adult patients, this high rate may reflect an increased rate of caries, or a high frequency of unmet dental need in this population.

A larger portion of patients (*n* = 18, 78%) received periodontal treatments. Periodontal treatment was also the overall most common type of treatment received in this population. Since all patients are routinely recommended for preventive periodontal care, this suggests that there is a gap in recommended preventive care for some patients in this study. This may contribute to increased rates of local and systemic inflammation in these patients and even to our highly observed restorative need. Patients also underwent frequent scaling and root planing procedures and periodontal maintenance, which are indicative of diagnosed periodontal disease. VEGF antagonists may also impact the healing of soft tissue via changes to signaling, which could have an adverse impact on healing from periodontal treatment. Although complications after periodontal therapy were not observed in this study, adverse outcomes and associated changes to treatment protocols should be considered in these patients as additional data becomes available.

Patients saw considerably fewer endodontic (*n* = 2, 3 total) and extraction (*n* = 12, 37 total) treatments. VEGF signaling plays a role in the development and vascularity of immature teeth, including contributing to healing [19]. Limited endodontic treatment experience in this population may suggest, however, that the impacts of vascularization and new vessel formation of VEGF antagonists do not compromise endodontic health, but this requires further investigation in the unique microenvironment of the pulp chamber and the impacts of VEGF signaling on healthy and diseased pulp tissue [20, 21].

Taken together, these findings suggest a gap in preventive care for patients taking VEGF agents. To date, no data exists on how VEGF antagonists and other biologic agents impact routine oral health maintenance. While there is an overall dearth of relevant information, including a lack of previous data on the dental disease experience or rates of disease on patients taking VEGF antagonists as well as minimal direct study of the conditions treated in these patients, there is no direct evidence available of a causative impact of the diseases treated in this population (macular degeneration, glaucoma, various primary and metastatic cancers) causing changes in dental status. Age related macular degeneration has been correlated with a possible increased rate of periodontal disease through observational studies, with age and chronic inflammation hypothesized as possible links between the two diseases [22, 23]. That being said, it is well documented that the treatment of cancers, including radiotherapy and chemotherapy, can cause severe dry mouth, leading to increased caries risk [24]. Furthermore, it was found that our cohort had high rates of comorbid diabetes mellitus, hypertension, and gastroesophageal reflux disease (GERD). It is known that diabetes is linked with increased rates of periodontal disease and poor wound healing [25, 26]. GERD has also been shown to lead to enamel erosion that can deteriorate tooth structure and function [27]. Given the possibility of impaired wound healing in this population, preventive health maintenance including periodontal maintenance may mechanistically be expected to positively contribute to oral health and prevention of disease in this population. However, further investigation is needed to examine any required adjustments to preventive strategies in this population, such as increased frequency of care or changes to delivery. Although this data is not compared to a control group, these rates begin to characterize the treatment experience in patients taking VEGF antagonists. Further characterization of this and other populations taking biologic agents, as well as comparison to matched groups, will allow for the identification of needs and risk profiles specific to these agents.

Across our population, the raw number and percentage of patients receiving dental treatment increased from 2017 to 2018, followed by decreases in 2019 and 2020 (Fig. 1; Table 3). In 2020, this can be explained by clinical limitations caused by the COVID-19 pandemic, while in 2019 there is no identifiable reason for limitation to treatment within this data. Following 2020, the percentages rebounded slightly, possibly illustrating an accumulated disease burden and demand for care, only to decrease again in 2022 and 2023. This decrease as time elapsed after the shutdowns associated with the COVID-19 pandemic may reflect existing and delayed treatment needs being appropriately addressed. In addition, treatment delivered in 2017 and 2023 is expected to be less than in the other years, since both included only six months of data.

Decayed, missing, and filled tooth data in this study allow for a first approximation of treatment needs over time in patients taking VEGF agents and a novel comparison to patients taking other biologic agents treated in the same setting. Data for patients taking VEGF antagonists were not significantly different than values for patients taking other biologic agents treated at Penn Dental Medicine, except missing teeth. Patients taking TNF-α Inhibitors had significantly fewer missing teeth than patients taking VEGF antagonists (3.88 vs. 8.10, *p* = 0.01). While this data is unable to elucidate the cause of any difference, the impact of VEGF antagonists on revascularization may compromise healing in patients taking these agents [28]. Alternatively, decreased immune infiltration in patients taking TNF-α antagonists may lead to a decreased immune response in this population [29]. As compared to nationally published data, patients in this study had fewer filled teeth (6.30 vs. 9.00, 0.01). While detailed understanding of the national cohort’s medical status is not available, the size and representative sampling suggests a mixed population. These direct medication effects require further investigation, and all results require confirmation in expanded cohorts to confirm their relevance.

Our study did not identify any post-operative complications, such as infections, pain, or need for additional treatments after primary dental care in our study population. However, similar studies have found complications after surgical treatments associated with VEGF antagonists. Studies looking at the incidence of adverse events following anti-VEGF intravitreal injections found both local and distant complications [30]. Systemic administration of VEGF antagonists has also been associated with many adverse effects, such as increased bleeding, cardiovascular events, delayed wound healing, and gastrointestinal issues, stemming from the interrelationship between antiangiogenic therapy and tumor microenvironments [31]. A related study by our group found no issues with healing but an increased rate of post-extraction pain in dental patients taking VEGF antagonists, possibly related to changes to pain modulation associated with these agents [32]. Compared to other types of Biologic Agents, patients on VEGF antagonist treatment saw the highest incidence of complications in this study, with 3 patients (out of 6) experiencing excessive pain after 4 of 5 extractions. The reasons behind this finding have not been fully explored to date, however related studies have found that there is a possible connection between rheumatoid arthritis and osteoarthritis and a dysregulation of VEGF 1 and 2 receptors [33, 34]. Furthermore, the literature has shown VEGF-A has an integral role in the “modulation of nociception and onset of chronic pain,” providing a possible explanation for post-operative complications associated with VEGF antagonist treatment [35]. The current findings of no clinical or symptomatic complications in this cohort continues to characterize the treatment experience of patients taking VEGF antagonists and can be compared to other findings in future studies on this population. Additional data will also allow for the establishment of best practices for any observed post-treatment complication in these patients and will allow for identification of procedure- or patient-based risk factors associated with complications in this population.

While this study provides novel insight into the oral status of patients taking VEGF antagonists, it does come with limitations. Our study only looked at the cohort of patients on VEGF antagonists at the University of Pennsylvania School of Dental Medicine and did not compare against a healthy control. In addition, it did not systematically compare to other populations taking biologic agents or to patients taking VEGF antagonists treated at other locations. Furthermore, the patient pool was limited to 23 patients across 7 years. As such, it is difficult to draw conclusions about the incidence of dental treatments amongst this group. The small sample size also precluded detailed analyses by underlying medical conditions or demographic factors. The results of this study serve as a descriptive baseline to provide early data on this population. Future studies should look to compare the frequency of dental treatments of patients taking VEGF antagonists (and other Biologic Agents) against healthy controls. Larger studies can expand the current findings and introduce the possibility of controlling for other patient factors. Expanded follow up in these patients may provide additional data including on long-term oral health outcomes of VEGF antagonists to guide treatment recommendations for these patients. Prospective studies of this population may also be used to investigate mechanistic connections and causative factors in dental treatment needs among patients taking VEGF antagonists.

## Conclusion

VEGF antagonists are a growing class of medications employed in various diseases including in the treatment of cancer. As such, it is imperative that the effects of these drugs on all aspects of health be elucidated. To date, the oral effects of these drugs have only begun to be explored. This study provides the first evaluation of dental status and needs in this population. Although our study did not find any complications in patients taking VEGF antagonists, our population was found to have a high level of restorative and periodontal treatment need, encompassing both development of dental caries and other treatment needs. Patients on VEGF antagonists and all other Biologic Agent types have a unique set of difficulties that complicate their care. As such, dental clinicians need to be aware of the needs of these patients and be prepared with proper treatment plans to accommodate them. To date, there are no such standardized guidelines. This study and studies similar aim to characterize this population to build a clinical understanding of how to safely and effectively treat these patients.

## Electronic supplementary material

Below is the link to the electronic supplementary material.

Supplementary Material 1

## Data Availability

All data will be available in deidentified fashion on reasonable request to the atuhors.
