# Supplementary material for: A retrospective, cross-sectional analysis of the dental status and needs of patients taking vascular endothelial growth factor (VEGF) antagonists
Source: Clin Oral Investig. 2025 Feb 22;29(3):151. doi: 10.1007/s00784-025-06230-7 (PMC11845543; doi:10.1007/s00784-025-06230-7)
Supplement: Supplementary file 1 — Supplementary Material 1 [file 784_2025_6230_MOESM1_ESM.docx]

**APPENDIX**

VEGF Antagonists searched for were aflibercept, ranibizumab, bevacizumab, ramucirumab, bevacizumab-awwb, bevacizumab-bvzr, avastin, Pravastin, alymsys, mvasi, vegzelma, zirabev, zaltrap, cyramza.

Restorative treatments were as follows: D2330-D2335, D2391-D2394, D2140-D2161, D2510-D2660, D2740-D2790, D2950, D2952-D2954, D2960-D2962. Periodontal treatments were as follows: D1110, D1120, D4910, D4341, D4342, D4200-D4300, D6010/D6010A, D4999. Endodontic treatments were as follows: D3110, D3120, D3220, D3310, D3320, D3330, D3346-D3348. Oral Surgical treatments were those in the range follows: D7XXX
